# Expression of novel extracellular sulfatases Sulf-1 and Sulf-2 in normal and osteoarthritic articular cartilage

**DOI:** 10.1186/ar2432

**Published:** 2008-05-28

**Authors:** Shuhei Otsuki, Noboru Taniguchi, Shawn P Grogan, Darryl D'Lima, Mitsuo Kinoshita, Martin Lotz

**Affiliations:** 1 Division of Arthritis Research, The Scripps Research Institute, 10550 North Torrey Pines Road, La Jolla, CA 92037, USA; 2 Department of Orthopedic Surgery, Osaka Medical College, 2–7 Daigaku-machi Takatsuki 569-8686, Osaka, Japan

## Abstract

**Introduction:**

Changes in sulfation of cartilage glycosaminoglycans as mediated by sulfatases can regulate growth factor signaling. The aim of this study was to analyze expression patterns of recently identified extracellular sulfatases Sulf-1 and Sulf-2 in articular cartilage and chondrocytes.

**Methods:**

Sulf-1 and Sulf-2 expressions in human articular cartilage from normal donors and patients with osteoarthritis (OA) and in normal and aged mouse joints were analyzed by real-time polymerase chain reaction, immunohistochemistry, and Western blotting.

**Results:**

In normal articular cartilage, Sulf-1 and Sulf-2 mRNAs and proteins were expressed predominantly in the superficial zone. OA cartilage showed significantly higher Sulf-1 and Sulf-2 mRNA expression as compared with normal human articular cartilage. Sulf protein expression in OA cartilage was prominent in the cell clusters. Western blotting revealed a profound increase in Sulf protein levels in human OA cartilage. In normal mouse joints, Sulf expression was similar to human cartilage, and with increasing age, there was a marked upregulation of Sulf.

**Conclusion:**

The results show low levels of Sulf expression, restricted to the superficial zone in normal articular cartilage. Sulf mRNA and protein levels are increased in aging and OA cartilage. This increased Sulf expression may change the sulfation patterns of heparan sulfate proteoglycans and growth factor activities and thus contribute to abnormal chondrocyte activation and cartilage degradation in OA.

## Introduction

Osteoarthritis (OA) is the most prevalent joint disease and is characterized by degradation of articular cartilage, subchondral bone remodeling, and joint inflammation [1,2]. Chondrocytes in OA cartilage are activated by cytokines and growth factors [3,4] to a catabolic phenotype that leads to progressive extracellular matrix (ECM) destruction and abnormal chondrocyte differentiation [4,5]. Cartilage ECM consists of collagens, glycoproteins, proteoglycans, and glycosaminoglycans (GAGs). The major GAGs in cartilage are hyaluronic acid, chondroitin sulfate, keratan sulfate, dermatan sulfate, and heparan sulfate. GAGs were previously shown to be important determinants of cartilage biomechanical properties but also have recently been shown to bind and regulate the activity of several cytokines and growth factors. In particular, the sulfation patterns of GAGs are critical in determining the binding capacity and specificity for cytokines and growth factors [6-9]. Heparan sulfate proteoglycans (HSPGs) also act as co-receptors for heparin-binding growth factors and cytokines [10]. The sulfation of heparan sulfate residues is required for interactions with heparin-binding factors that are also know to be important regulators of chondrocytes, including Wnt, fibroblast growth factor (FGF), vascular endothelial growth factor (VEGF), and bone morphogenetic proteins (BMPs) [11-14].

Sulfotransferases and sulfatases establish GAG sulfation in the endoplasmatic reticulum and Golgi network prior to secretion [15]. Classic sulfatases are intracellular enzymes that cleave sulfate esters from substrates that range from small cytosolic steroids, such as estrogen sulfate, to complex cell surface carbohydrates, such as the GAGs [15]. A novel class of extracellular heparan sulfate 6-O endosulfatase (Sulf) has recently been identified and in mammalians includes two isoforms, Sulf-1 and Sulf-2 [16-18]. These enzymes exist in a cell surface-associated and soluble form and hydrolyze the 6-O sulfate of HSPGs [17,19]. Most of the current information on Sulf-1 and Sulf-2 is related to cancer and development [20-23]. Specifically, 6-O sulfation of heparan sulfate is required for receptor dimerization and FGF signaling while 6-O desulfation is associated with reduced FGF2 signaling [24]. Sulf-1 also regulates Wnt signaling through desulfation of cell surface HSPGs [16].

OA is associated with changes in GAG expression levels and sulfation patterns [6,8,9], but mechanisms and consequences remain to be analyzed. This study addresses the hypothesis that the novel extracellular sulfatases may be involved in regulating the growth factor signaling balance in articular cartilage. The results show that Sulf-1 and Sulf-2 are (a) expressed in human articular cartilage, and (b) are preferentially expressed in the superficial zone and that (c) their expression is altered in osteoarthritic and aging cartilage.

## Materials and methods

### Cartilage procurement and processing

All tissue samples were graded according to a modified Mankin scale [25], with a score of less than 3 points being normal and a score of greater than 5 representing OA [26]. Normal articular cartilage was harvested from femoral condyles and tibial plateaus of human tissue donors under approval of the Scripps Human Subjects Committee. Osteoarthritic cartilage was obtained from patients undergoing knee replacement surgery. The thickness of these cartilages ranged from 1.5 to 2.8 mm. Once cartilage surfaces were rinsed with saline, scalpels were used to cut parallel sections 5 mm apart, vertically from the cartilage surface onto the subchondral bone. These cartilage strips were then resected from the bone. Human chondrocytes were isolated and cultured as described previously [27]. The cartilage tissue was incubated with trypsin at 37°C for 10 minutes. After the trypsin solution was removed, the tissue slices were treated for 12 to 16 hours with type IV clostridial collagenase in Dulbecco's modified Eagle's medium (DMEM) with 5% fetal calf serum. After initial isolation, the cells were kept in high-density cultures in DMEM (high glucose) supplemented with 10% CS, L-glutamine, and antibiotics and allowed to attach to the surface of the culture flasks. After the cells had grown to confluence, they were split once (passage 1) and grown to confluence again for use in the experiments.

### RNA isolation from cartilage and cultured chondrocytes

RNA was isolated from fresh frozen cartilage by homogenizing the tissue in a freezer mill (Spex CertiPrep, Inc., Metuchen, NJ, USA) and extracting the homogenate in Trizol (Life Technologies, Inc., now part of Invitrogen Corporation, Carlsbad, CA, USA). The samples were extracted with chloroform and centrifuged at 15,000 *g *for 20 minutes, and the aqueous phase was collected. An equal volume of 70% ethanol was added, mixed, and applied to RNeasy columns (Qiagen Inc., Valencia, CA, USA). RNA concentrations were determined using RiboGreen reagent (Molecular Probes Inc., now part of Invitrogen Corporation). Total RNA was isolated from chondrocyte cultures plated at confluence at 3 × 10^6 ^cells per 100-mm plate using the RNeasy kit (Qiagen Inc.) with on-column DNA digestion. Complementary DNA was produced using the SuperScript III First-Strand kit (Invitrogen Corporation) with random hexamers.

### Quantitative polymerase chain reaction for Sulf-1 and Sulf-2

Sulf-1 and Sulf-2 primers and conditions for reverse transcription-polymerase chain reaction (RT-PCR) were based on the protocol of Morimoto-Tomita and colleagues [17]. Real-time RT-PCR with SYBR green detection was performed using an iCycler (Bio-Rad Laboratories, Inc., Hercules, CA, USA) as follows: 2 minutes at 50°C and then 10 minutes at 95°C for initial denaturation, followed by 40 cycles at 95°C (15 seconds), 60°C (1 minute), followed by the measurement of fluorescence at the end of each cycle. Each run included a melting curve to determine the correct response of the primers [28]. The following primers were used: Sulf-1: forward 5'-AGACCTAAGAAT CTTGATGTTGGAA-3', reverse 5'-CCATCCCATAACTGTCCTCTG-3'(74 base pairs [bp], NM15170), Sulf-2: forward 5'-TGAGGGAAGTCCGAGGTCAC-3', reverse 5'-CTTGCGGAGTTTCTTCTTGC-3' (194 bp, NM018837, NM198596), glyceraldehyde-3-phosphate dehydrogenase (GAPDH): forward 5'-ACCCACTCCTCCACCTTTGA-3', reverse 5'-ATGAGGTCCACCACCCTGTT-3'.

Primers were selected in consideration of the low homology between the sequences of Sulf-1 and Sulf-2. Furthermore, human Sulf-2 primers were designed to detect both Sulf-2 splice variants, NM 018837 and NM 198596. The specificity of detection of Sulf-1 and Sulf-2 was confirmed by sequencing the PCR products after isolation with the QIAquick gel extraction kit (Qiagen Inc.). Changes in Sulf gene expression were calculated relative to GAPDH.

### Histology and immunohistochemistry

Cartilage tissues were fixed with 4% paraformaldehyde and stained with safranin O. Sulf antibodies were purchased from Abcam Inc. (Cambridge, MA, USA). Paraffin-fixed samples were first deparaffinized in xylene substitute Pro-Par Clearant (Anatech Ltd., Battle Creek, MI, USA), ethanol then water for rehydration. After washing with PBS, sections were blocked with 0.1% Tween20 with 3% normal goat serum for 30 minutes at room temperature. Sulf-1 and Sulf-2 antibodies (2 μg/mL) and normal mouse IgG (1 μg/mL) as negative control were applied and incubated overnight at 4°C. After washing with PBS, sections were incubated with biotinylated goat anti-mouse secondary antibody for 30 minutes (1:200; Vector Laboratories Inc., Burlingame, CA, USA) and then incubated with Vectastain ABC-AP kit (AK-5000; Vector Laboratories Inc.) for 30 minutes at room temperature. Finally, sections were stained with an alkaline phosphatase substrate kit (Vector Laboratories Inc.).

### Quantification and localization of signals throughout cartilage

Sulf-1 and Sulf-2 localization throughout each cartilage zone was assessed systematically by counting positive and negative cells in a 50 × 50 μm grid (using a ×40 field objective) starting from the cartilage surface to the deep zone. This was repeated a minimum of five times for each section. The identification of each zone was based on previously reported characteristics that comprise cell shape, morphology, orientation, and pericellular matrix (PM) deposition [29]. Thus, superficial zone (SZ) cells were characterized by their elongated shape, their parallel orientation relative to the surface, and lack of extensive PM. These cells predominate within the first 50 μm. The middle zone (MZ) was distinguishable by rounded cells that did not exhibit an organized orientation relative to the surface, that have ECM rich in proteoglycans, and that show the presence of PM. Conversely, deep zone (DZ) cells were recognized with an extensive PM deposition and organized in columns of chondron groups of three or more cells. The depth of each zone was recorded for each section for comparative analysis on the frequency of positive signal in each zone. The frequency of positive cells was expressed as a percentage relative to the total number of cells counted in each zone.

### Western blotting

Cartilage was cut into 1-mm-thin slices, and 200 to 1,000 mg of frozen cartilage was pulverized in a liquid nitrogen-cooled freezer mill for two cycles of 1.5 minutes at the rate of maximum impact frequency. Dry weight of normal and OA cartilage was measured and the same amount of protein was resuspended in SDS gel loading buffer (50 mM Tris pH 6.8, 10% glycerol, 4% sodium dodecyl sulfate, 10% 2-mercaptoethanol, and 0.001% bromophenol blue) and mixed for 2 hours at room temperature. Centrifugation at 14,000 rpm was performed for 30 minutes and then supernatants were harvested and heated at 80°C for 10 minutes. The concentrated samples were then adjusted for equal volumes before resolution on 12% Tris-Glycine gels (Invitrogen Corporation). Protein was transferred to nitrocellulose membranes (Invitrogen Corporation), blocked with 5% dry milk in Tris-buffered saline–Tween (TBST), and blotted with mouse polyclonal antibody specific for Sulf-1 or Sulf-2 (Abcam Inc.) for 1 hour. The membranes were then incubated with horseradish peroxidase-conjugated anti-mouse IgG (Santa Cruz Biotechnology, Inc., Santa Cruz, CA, USA) for 1 hour. Afterwards, the membranes were washed three times with TBST and developed using the enhanced chemiluminescent substrate from Pierce (Rockford, IL, USA).

### Analysis of murine joints

All animal experiments were performed according to protocols approved by the Institutional Animal Care and Use Committee at The Scripps Research Institute (La Jolla, CA, USA). Sulf-1 and Sulf-2 expression was analyzed by immunohistochemistry in temporomandibular (TM) joints and knee joints of 1-, 6-, 9-, and 12-month-old C57BL/6J mice. Each mouse joint was cut in half along the mid-sagittal plane and fixed in 10% zinc-buffered formalin (Z-Fix; Anatech Ltd.) for 2 to 3 days and then decalcified in Shandon TBD-2 decalcifier (Fisher Scientific Pittsburgh, PA, USA) for 2 to 3 weeks. Three-millimeter serial sections (from posterior to anterior) were cut and immunostained for Sulf-1 and Sulf-2 as described above.

### Statistical analysis

Statistically significant differences between two groups were determined with *t *tests. The results are reported as mean ± standard deviation. *P *values of less than 0.05 were considered significant.

## Results

### Sulf gene expression in articular cartilage

**Figure 1:**
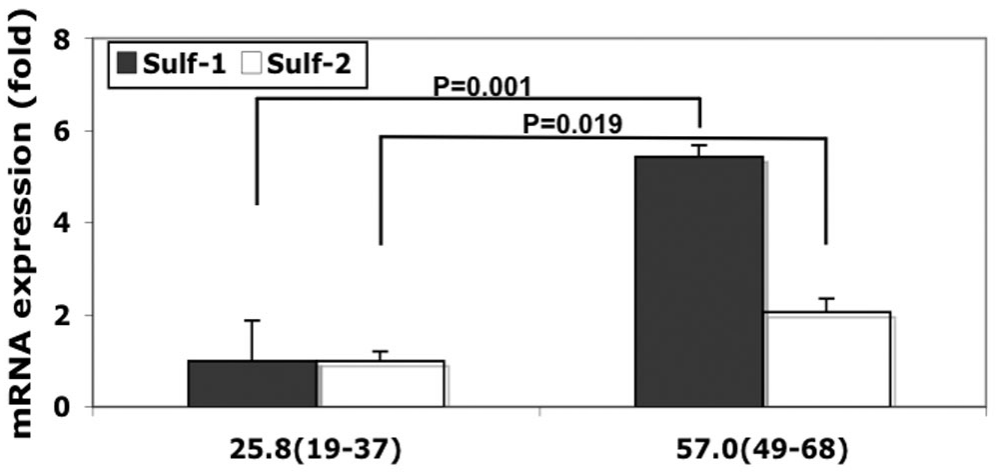
Sulf mRNA expression in normal and osteoarthritis (OA) cartilage. Sulf-1 and Sulf-2 mRNA expression in articular cartilage were determined by quantitative polymerase chain reaction in eight normal (mean age: 20.3 years, range: 19 to 37 years; Mankin score: 0 to 2 points) and eight OA (mean age: 57 years, range: 49 to 68 years; Mankin score: 7 to 10 points) donors. Both Sulf-1 and Sulf-2 expression were significantly higher in the OA group (Sulf-1: *P *= 0.001, Sulf-2: *P *= 0.019).

Sulf-1 and Sulf-2 mRNA expression in eight OA donors (49 to 68 years old; Mankin score: 7 to 10 points) was significantly higher than in eight young donors (19 to 37 years old; Mankin score: 0 to 2 points) as determined by real-time PCR (Figure 1).

### Localization of Sulf-1 and Sulf-2 proteins in human articular cartilage

**Figure 2:**
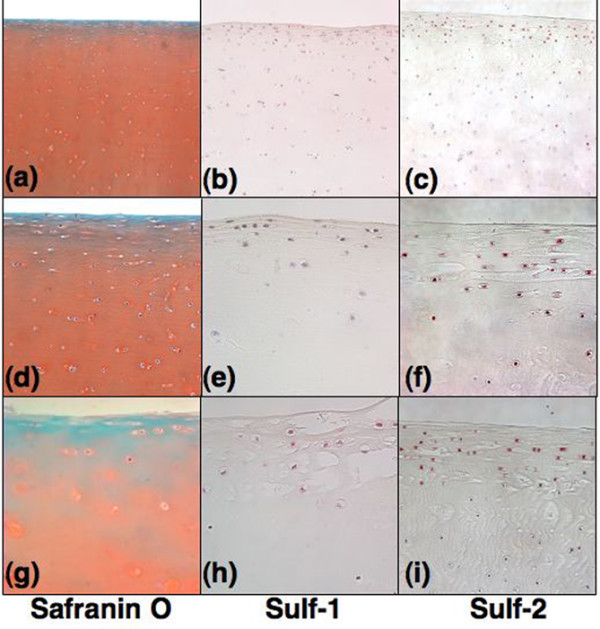
Localization of Sulf-1 and Sulf-2 in normal cartilage. Representative sections of 26-year-old **(a, d) **and 74-year-old **(g) **normal cartilage (Mankin scores: 0 and 2) as seen on safranin O staining are shown (n = 8; 19 to 37 years old). Sulf-positive cells (brown staining) are present in the superficial zone and the top of the middle zone, and Sulf-2 expression is greater than Sulf-1 **(b, c, e, f, h, i) **in both young and old cartilage. Magnifications: ×10 **(a-c) **and ×40 **(d-i)**.

**Figure 3:**
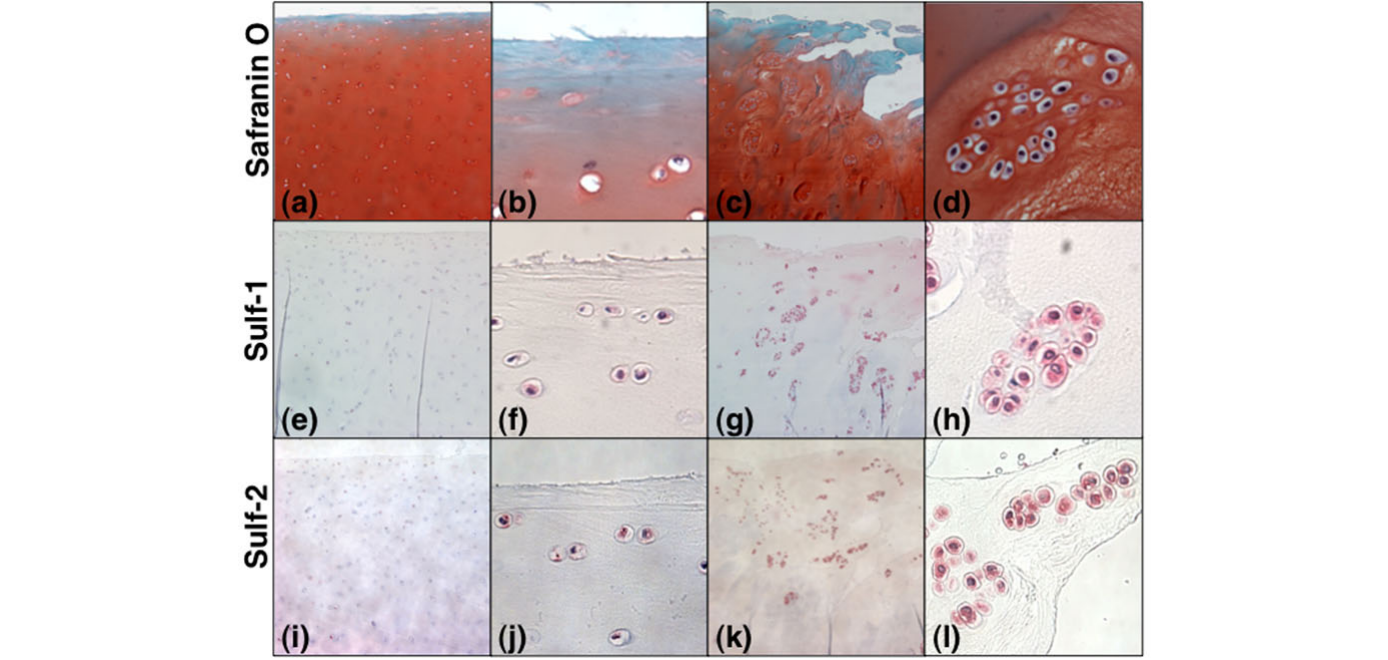
Localization of Sulf-1 and Sulf-2 in normal-appearing and fibrillated cartilage from the same osteoarthritis (OA) donor. Sulf localization was determined with 16 donors (19 to 82 years old). Cartilage from a representative 65-year-old donor had both normal areas (Mankin score: 2) and OA areas (Mankin score: 8). Sulf-positive cells were more frequent in OA areas than in normal-appearing cartilage. Magnifications: ×10 **(a, c, e, g, i, k) **and ×100 **(b, d, f, h, j, l)**.

Young and old normal samples as seen on safranin O staining (Figure 2a, d, g) had only a few Sulf-positive cells in the superficial zone (Figure 2b, c, e, f, h, i) and no positive cells in the middle and deep zones. In general, the expression of Sulf-2 appeared more intense than Sulf-1 in normal cartilage. In OA cartilage, many positive cells were detected, especially in chondrocyte clusters (Figure 3g, h, k, l). The representative example of 65-year-old cartilage had both normal areas (Mankin score: 2) (Figure 3a, b) and OA areas with fibrillations and cluster formation (Mankin score: 8) (Figure 3c, d). The normal appearing areas from OA joints had 18.5% Sulf-1-positive and 31.9% Sulf-2-positive cells in the superficial zone (Figure 3e, f, i, j), which was greater than in normal cartilage (Figure 2). On the other hand, OA areas had 75.3% Sulf-1-positive and 73.2% Sulf-2-positive cells (Figure 3g, h, k, l).

**Figure 4:**
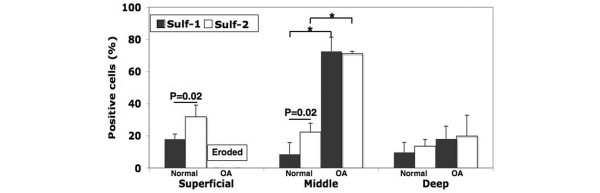
Sulf-1 and Sulf-2 expression in specific zones of normal and osteoarthritis (OA) cartilage. The number of Sulf-1- or Sulf-2-positive cells was counted in the superficial, middle, and deep zones of sections from normal (n = 8) and OA (n = 8) cartilage that were stained with specific antibodies. In normal cartilage, the percentage of Sulf-2-positive cells was highest in the superficial zone. The superficial zone in OA cartilage was eroded. The OA middle zone had significantly more Sulf-1-positive cells than the other zones (*P < 0.01). Sulf-2 expression in normal cartilage was significantly higher than Sulf-1 (*P *= 0.02).

Figure 4 shows quantitative analysis of the zonal distribution of Sulf-1- and Sulf-2-expressing cells in eight normal (17 to 37 years old) and eight OA (43 to 82 years old) donors. In OA, the superficial zone was already eroded. The middle zone in OA cartilage had significantly more positive cells than normal (*P < 0.01). Moreover, the number of Sulf-2-positive cells in the superficial and middle zones was greater than Sulf-1-expressing cells (*P *= 0.02).

**Figure 5:**
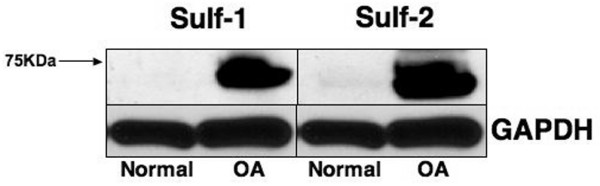
Sulf-1 and Sulf-2 protein expression in normal and osteoarthritis (OA) cartilage. Sulf protein expression in normal (43 years old; Mankin score: 2) and OA (79 years old; Mankin score: 9) cartilage. Immunoblottings of Sulf-1, Sulf-2, and GAPDH (glyceraldehyde-3-phosphate dehydrogenase) were performed on protein extracts from normal and OA cartilage.

Western blotting was performed to visualize Sulf proteins and determine differences in the expression between normal and OA. In total protein extracts from normal cartilage, Sulf-1 and Sulf-2 were not detectable. In contrast, high levels of Sulf-1 and Sulf-2 protein were detected in OA cartilage (Figure 5). The major Sulf-1 and Sulf-2 protein bands migrated at approximately 72 kDa, which is the molecular mass of the secreted proteins [17,30,31].

### Sulf-1 and Sulf-2 expression in murine joints

**Figure 6:**
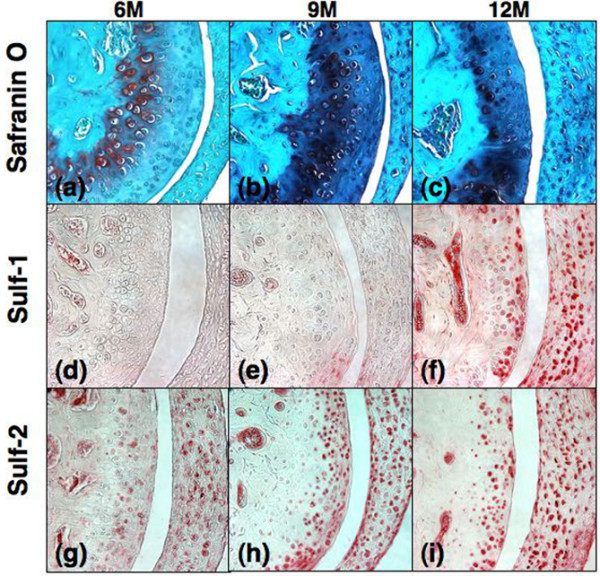
Sulf-1 and Sulf-2 expression in murine temporomandibular joints. Safranin O staining **(a-c) **and immunohistochemistry **(d-i) **were performed on sections from temporomandibular joints of C57BL/6J mice at 6, 9, and 12 months of age (n = 6). Magnification: ×40.

**Figure 7:**
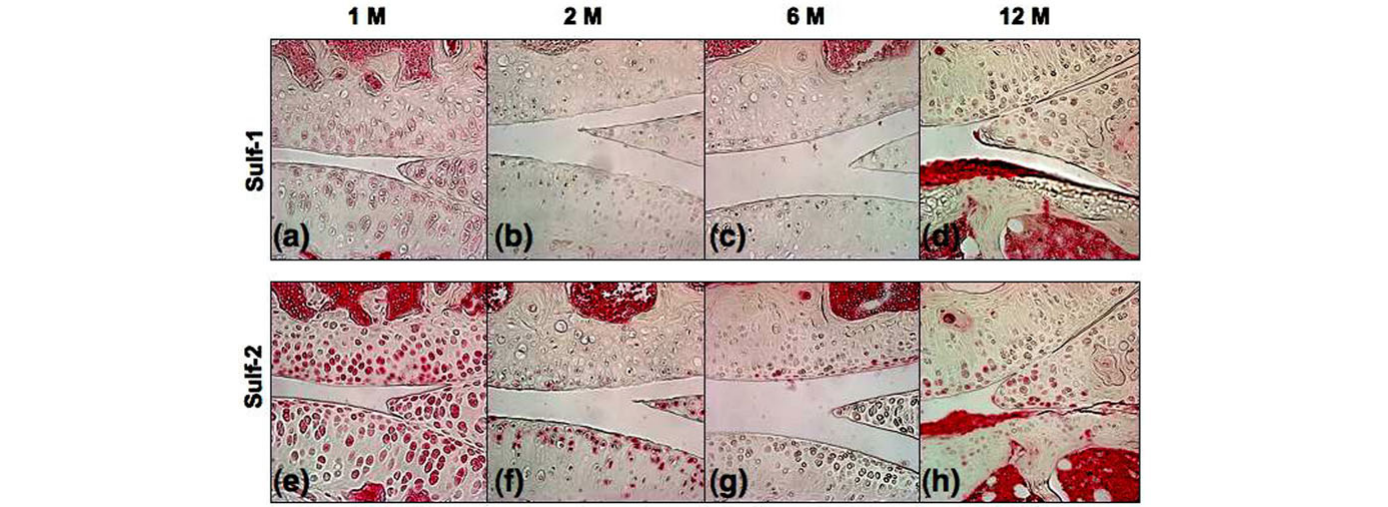
Sulf-1 and Sulf-2 expression in murine knee joints. Immunohistochemistry for Sulf-1 **(a-d) **and Sulf-2 **(e-h) **was performed on sections from knee joints of C57BL/6J mice at 1, 2, 6, and 12 months of age (n = 8). Magnification: ×40.

TM joints from normal C57BL/6J mice (n = 6) were analyzed with safranin O staining (Figure 6a–c) and immunohistochemistry for Sulf-1 and Sulf-2 (Figure 6d–i). Histology showed thinning and reduced cell density in articular cartilage with increasing age (Figure 6c). In 6-month-old mice, only a few cells were positive for Sulf-1 but Sulf-2-positive cells were present throughout the cartilage. There was a marked increase in Sulf-2 expression at 9 months and in Sulf-1 expression at 12 months. In murine knee joints (n = 8), there was high Sulf expression at 1 month of age, followed by a decrease with joint maturation. Increased expression of Sulf-1 and Sulf-2 was seen in the articular cartilage of murine knee joints by 12 months of age, when early OA-like changes become apparent (Figure 7).

## Discussion

Chondrocytes in osteoarthritic cartilage are activated by cytokines, growth factors, and mechanical stress to produce matrix-degrading enzymes and pro-inflammatory cytokines with an overall shift from anabolic to catabolic responses [32,33]. Besides control of gene expression, protein synthesis, and secretion, the biological activity of cytokines and growth factors is regulated by binding to ECM proteins such as GAGs [34,35]. The sulfation pattern of HSPGs has recently been shown to be critical for determining the specificity and affinity of binding to growth factors and morphogens [36]. The sulfation patterns of HSPGs are determined during intracellular biosynthesis and can be further modified on cell surface-associated and extracellular HSPGs by a novel class of extracellular sulfatases which includes two enzymes, Sulf-1 and Sulf-2. Previously, Sulf-1 and Sulf-2 mRNAs were shown to be expressed at high levels in regions of developing cartilage and bone [37]. The present study reports on the expression of Sulf in mature cartilage and changes with aging and OA.

In normal articular cartilage, Sulf-1 and Sulf-2-positive cells were predominantly localized in the superficial zone and Sulf-2 was more highly expressed than Sulf-1. This observation adds further to the zone-specific differences of chondrocyte subsets, in particular of the superficial zone cells [38-40].

OA cartilage showed higher expression of Sulf-1 and Sulf-2 as compared with normal tissue in all experimental approaches used in the present study, including quantitative PCR on cartilage and cultured chondrocytes, immunohistochemistry, and Western blotting. Aging and OA are closely linked. To address the influence of these variables on Sulf expression, we analyzed normal-appearing and fibrillated cartilages in the same joints from patients with OA. In the OA joints, Sulf expression was higher in the fibrillated areas. Even in areas that had almost normal surface layers and safranin O staining patterns, Sulf expression was higher than in normal cartilage from young healthy donors. The TM joint is an important growth and articulation center in the craniofacial complex, and with aging, it develops spontaneous degenerative OA lesions [41]. TM joints showed strongly increased Sulf expression between 6 and 12 months of age, when cartilage thickness and cellularity were reduced, but fibrillations had not yet developed. Sulf expression was also determined in murine knee joints. Interestingly, Sulf expression was high at 1 month of age and decreased with joint maturation, suggesting a role in this process. By 12 months of age, Sulf expression increased again with the simultaneous development of OA-like changes. Taken together, these findings from human and murine joints indicate that Sulfs are upregulated with age and at early stages of the matrix degradation process.

The present observations of increased Sulf expression suggest a role in OA pathogenesis. Altered GAG sulfation patterns on chondroitin sulfate and dermatan sulfate have been reported in aging and OA [9,42], but changes in heparan sulfation patterns under these conditions have not yet been analyzed. The HSPGs are potential targets of Sulf [19], and their expression in articular cartilage and changes in OA have been demonstrated in several previous publications on syndecan [43-49], perlecan [50-54], and glypican [44,54]. In particular, syndecan-1, syndecan-3 [45,46], and perlecan [50] are overexpressed in severe OA. Furthermore, some of these studies have shown that HSPGs are overexpressed, specifically in cell clusters in OA cartilage. In this study, we also showed Sulf-1 and Sulf-2 overexpression in OA cartilage, particularly in clusters. Collectively, this information documents the presence of the HSPGs that are the major known sulfatase substrates in articular cartilage. In addition, there appears to be similar expression of the enzymes and substrates in OA-affected cartilage. Changes in sulfation of heparan sulfate are important in cell behavior and organogenesis [55] and affect several growth factor signaling pathways. 6-O sulfated heparan sulfates are required for FGF receptor dimerization. Sulf-1 desulfates cell surface heparan sulfate and inhibits FGF signaling [24,56]. Im and colleagues [57] showed that FGF2 induced matrix metalloproteinase-13 in articular chondrocyte and contributes to OA progression. FGF2 may regulate Sulf expression and maintain the anabolic and catabolic balance in cartilage.

Sulf-1 also mediates 6-O desulfation of the heparan sulfate-Wnt complex so that it interacts with Frizzled receptor, initiating Wnt target gene expression [19]. Wnt signaling is important in cartilage. Wnt and β-catenin activation are associated with inhibition of type II collagen expression [58] with GAG loss [8] and abnormal chondrocyte differentiation in OA [59]. Thus, Wnt signaling, activated by Sulf, may accelerate the progression of OA. Sulf-1 regulates BMP signaling, which is important in cartilage homeostasis. The BMP antagonist Noggin is a heparin-binding protein that is associated with the cell surface through HSPGs, where it inhibits BMP signaling. Sulf-1 desulfates heparan sulfate, releases Noggin, and thus restoring BMP signaling [11].

## Conclusion

This study is the first to show increased Sulf expression in OA cartilage. Sulf-1 and Sulf-2 are highly expressed in OA cartilage, especially in clusters and even in normal-appearing cartilage in OA joints. The ability of Sulfs to regulate growth factor pathways (such as FGF, Wnt, or BMP) that are important in cartilage suggests that their overexpression in OA contributes to the abnormal chondrocyte activation and ECM degradation. Inhibition of Sulfs may represent a new approach to correct these pathogenetic processes.

## Abbreviations

BMP = bone morphogenetic protein; bp = base pairs; DMEM = Dulbecco's modified Eagle's medium; ECM = extracellular matrix; FGF = fibroblast growth factor; GAG = glycosaminoglycan; GAPDH = glyceraldehyde-3-phosphate dehydrogenase; HSPG = heparan sulfate proteoglycan; OA = osteoarthritis; PCR = polymerase chain reaction; PM = pericellular matrix; RT-PCR = reverse transcription-polymerase chain reaction; Sulf = heparan sulfate 6-O endosulfatase; TBST = Tris-buffered saline-Tween; TM = temporomandibular.

## Competing interests

The authors declare that they have no competing interests.

## Authors' contributions

SO carried out the experimental work, performed the statistical analysis, and drafted the manuscript. NT and SG performed experimental work and helped to draft the manuscript. DD'L and ML analyzed the data. ML designed and organized the study and drafted the manuscript. All authors read and approved the final manuscript.
